# Regulatory Aspects of Allergen-Specific Immunotherapy: Europe Sets the Scene for a Global Approach

**DOI:** 10.1097/WOX.0b013e318272484e

**Published:** 2012-10-15

**Authors:** Sergio Bonini

**Affiliations:** 1 Department of Medicine Second University of Naples and Institute of Translational Pharmacology Italian National Research Council, Rome Italy

## 

After 100 years from its introduction in the treatment of allergic patients, [1-3] regulatory aspects of allergen-specific immunotherapy (ASI) are still incompletely defined and very heterogeneous among different countries.

To better understand the scenario of regulatory documents applying to ASI in Europe and their different compelling value, it might be useful, for pure didactic purposes of this article and in agreement with the thoughtful paper of Kaul et al, [4] to classify them according to a hierarchic scale (from A to D), as in the Shekelle ranking of the level of evidence of scientific statements [5].

*Type A *documents are represented by European Community (EC) Regulations, which are valid and compelling for all Member States. The same value can be assigned to the European Pharmacopeia, which includes a specific Monograph on Allergen Products.

Among *Type B *documents, we can include EC Directives. These are not compelling for Member States, which however are obliged to adapt to the Directives by producing national laws within a given period.

*Type C *documents may be represented by the European Medicine Agency (EMA) Guidelines. These have only value of orientation, mainly for clinical trials (which should however justify, in case of discrepancies, why guidelines were not followed). A special type of guideline is represented by the International Conference on Harmonization of Technical Requirements for Registration of Pharmaceutical for Human Use (ICH), a consensus document agreed on by the European, American, and Japanese Regulatory Agencies.

Position Papers or Practice Parameters of Expert Panels or of Scientific Societies [6-11] can be considered as *Type D *documents because they only represent individual opinions, even if evidence based, certainly useful for clinical practice but of no regulatory value.

As in the Shekelle classification, the different types of evidence imply a corresponding level of strength of recommendations, documents on immunotherapy have a different regulatory impact, mandatory for European Regulations and National Laws following EC Directives, of orientation only for Types C and D documents.

Table 1 reports some examples of regulatory documents for IAS distinguished according to the categories described above.

**Table 1:** Types of Regulatory Documents for Allergen-Specific Immunotherapy

Type	Examples
**A**	
	• Regulation No. 726/2004/EC laying down Community Procedures for the authorization and supervision of medicinal products for human and veterinary use and establishing a EMA. OJ 2004;136:1-33. • Regulation No. 1901/2001/EC on medicinal products for pediatric use and amending Regulation EEC No. 1768/92, Directive 2001/83/EC, and Regulation No. 726/2004/EC OJ 2006;378:1-19. • European Pharmacopeia, Monograph on allergen products. 01/2010:1063
**B**	
	• Directive 2001/83/EC of the European Parliament and of the Council on the community code relating to medicinal products for human use. OJ 2004;1,341:67-112. • Directive 2001/20/EC of the European Parliament and of the Council on the approximation of the laws, regulations, and administrative provisions of the Member States relating to the implementation of good clinical practice in the conduct of clinical trials on medicinal products for human use. OJ 2001;1,121:24-44. • Commission Directive 2003/63/EC amending Directive 2001/83/EC. OJ 2003/1.159:46-94. • Commission Directive 2003/94/EC laying down the principles and guidelines of good manufacturing practice in respect of medicinal products for human use and investigational medicinal products for human use. OJ 2003;262:22-26.
**C**	
	• **EMA Guideline on Allergen Products: Production and Quality Issues. 2008. EMEA. CHMP/BWP/30483!/2007** • *Referring to both allergen extracts (and their classification in homologous groups) and allergens produced trough recombinant technology* • EMA Note for Guidance on Preclinical Pharmacological and Toxicological Testing of Vaccines. 1998/CPMP/SWP/465/95 • ICH Topic M3 (R2). Note for guidance on non-clinical safety studies for the conduct of Human clinical trials and marketing authorization for pharmaceuticals. 2009. CPMP/ICH/286/95 • ICH Topic E6 (R1). Note for guidance on GPC. 2002.CPMP/ICH/135/95 • ICH Topic E9. Note for guidance on statistical principles for clinical trials. 1998.CPMP/ICH/135/95 • Points to consider on application with: 1. Meta-analyses; 2. One pivotal study. 2001.CPMP/EWP/2330/99 • **EMA Guideline on the Clinical Development of Products for SIT for the treatment of allergic Diseases. 2008.CHMP/EWP/18504/2006** • **EMA/PDCO Standard Paediatric Investigation Plan for Allergen Products for Specific Immunotherapy. EMA/PDCO/737605/2009. Revision 2, 3-3-2010**.
**D**	
	• Allergen immunotherapy: therapeutic vaccines for allergic diseases. A WHO Position Paper [6]
	• Recommendations for standardization of clinical trials with allergen-specific Immunotherapy for respiratory allergy. A statement of the World Allergy Organization Task Force [7] • Sublingual immunotherapy. WAO Position Paper [8] • EAACI Immunotherapy Position Paper [9] • Allergen immunotherapy. A practice parameter. Third Update [10] • Allergen immunotherapy in children. An EAACI position statement [11] • The CONSORT statement checklist in allergen specific immunotherapy. A GA2LEN paper [12,13]

## The Present

Allergen products have been marketed for many years in Europe as named patient products (NPP), following a marketing authorization, which had only to answer to Good Manufacturing Practice requirements. Accordingly, the pharmacovigilance system for ASI is poorly structured, being only based on the voluntary reporting of side effects by doctors and patients. This generated from one side a diffuse skepticism among nonallergy specialists on the efficacy of the treatment--despite the results of several meta-analysis of Randomized Clinical Trials (RCTs) after the original one published in 1954 [14] --as well as a perhaps excessive and unjustified alarm on its safety, particularly in England.

In Europe, a crucial turning point in the regulatory aspects of ASI has been produced by the Directive 2001/20/EC and its amending Directive 2003/63/EC. These Directives state 2 fundamental issues for allergen products used both for in vivo diagnostics and for ASI of allergic diseases:

1. Allergens are medicines because they are capable to identify or to induce an acquired change in the immune response to a sensitizing agent.

2. As medicines produced with an industrial process, allergens therefore require, in Europe, a marketing authorization according to the procedures established for all drugs (ie, centralized, mutual recognition, decentralized, or national [15-18]) and following a clinical development through all phases of RCTs.

In fact, the Directive 2001/83 also establishes that in special circumstances, NPP may be prescribed for individual patients under the direct doctor responsibility. This exception is responsible for the different behaviors adopted in different European countries, depending on the national legislation and on the prevalent use of NPP versus registered allergen products [4,19].

The EMA and the new Executive Director (see box) is now considering to come, through a discussion open to all stakeholders including the World Allergy Organization (WAO) and the European Academy of Allergy and Clinical Immunology EAACI, to a more uniform approach in Europe on ASI, which might also represent the basis for a more global consensus with other regulatory agencies.

## The Future

The high prevalence of allergic diseases and their chronic coursed--that are responsible for high costs for both individuals and the Society--should give to ASI a high priority in the agenda of all national and international regulatory bodies. Furthermore, because allergic diseases are a global problem, national approaches should be harmonized, to avoid, for instance, the discrepancies at present observed between Europe and the United States [20-22] and to allow a uniform standard of care in any part of the world.

Of course, different economic resources and health priorities among countries may certainly influence access to AIS and reimbursement policies. However, the same efficacy and safety requirements should be assured to any citizen independently from the place of residence, also taking into account the actual high degree of mobility.

There is no doubt that ASI products should be supported by adequate preclinical and clinical dossiers for both adults and children, as for all other drugs. This will imply a hard commitment for both allergen manufacturers and research scientists in providing high-quality allergens, defining adequate study designs, selecting distinct phenotypes of patients on the basis of reliable clinical and biological markers, [23,24] identifying clinically relevant objective and patient-related outcome measures, such as quality-of-life outcome measures. These should be selected depending on which of the 4 potential claims of ASI that, according to EMA Guidelines, should receive support in future clinical trials:

• Treatment of allergic symptoms: efficacy in short-term clinical trials in the first pollen season or, in perennial allergies, after some months of treatment;

• Sustained clinical effect: maintenance of significant and clinically relevant efficacy during 2 to 3 treatment years;

• Long-term efficacy and disease modifying effect: sustained significant and clinically relevant efficacy in post-treatment years;

• Curing allergy: sustained absence of allergic symptoms in posttreatment years.

Clinical trials in children should follow the EMA Standard Pediatric Investigation Plan. However, this has possibly to be reconsidered, to allow the feasibility and ethics required for studies in this particular population sample.

Finally, outcome measures of future clinical trials should also include pharmacoeconomic end points that may allow a benefit/risk assessment, particularly for the most severe forms of allergic diseases still waiting for adequate treatment.

It is important to note that regulatory issues of ASI will certainly reflect on the rights of patients to have access to an optimal heath care and on a core competence of allergists. In fact, it can be expected from one side that a reduced number of allergen products will enter and pass the long and expensive process of registration and from the other side that products obtaining a marketing authorization will be more easily available for prescription to other specialists. Accordingly, a specialty based on skin-prick testing aimed at ASI prescription, in the lack of specific irreplaceable competences, will have no more reasons to survive the unavoidable cuts of the various national health systems. On the other hand, rare and severe allergies may still need specialized diagnostic approaches and availability of orphan products. The new Allergy specialist should therefore adapt to the changing health needs, hear the last call not to forget to be also a Clinical Immunology specialist, and enlarge his/her competence to the growing field of Biologicals.

In conclusion, the second Century of ASI may open a new era for all stakeholders in the area of Allergy and Clinical Immunology, [25] which hopefully may reflect on a better management of the increasing number of patients with allergic and immunologic diseases.

## End notes

Professor Guido Rasi has taken over as Executive Director of the European Medicine Agency (EMA) on November 16, 2011.

Professor Guido Rasi obtained his doctor of medicine at the University of Rome "La Sapienza," where he became Specialist in Internal Medicine in 1983 and in Allergology and Clinical Immunology in 1986.

In 2008, Professor Rasi became professor of Clinical Microbiology at the University of Rome Tor Vergata.

From July 2008 to 2011, he was general director of the Italian Drug Agency. During his mandate, he promoted the funding of the Agency for several projects of independent research in the area of asthma, allergic/immunologic diseases, immunotherapy, and biologicals (*Eur J Clin Invest*. 2010;40:69-86).

On September 7, 2011, the European Parliament approved his nomination by the European Medicines Agency's Management Board as EMA Executive Director for the period 2011 through 2016.

Research activity of Professor Rasi in the area of allergy and clinical immunology includes studies (performed in collaboration with Sergio Bonini, Tom Platts Mills, Bruce Mitchell, and Martin Chapman) on the influence of environmental factors (infectious agents, smoking) on IgE production and on the genetic effect in atopic twins on the serum levels of IgE and IgG4 antibodies to major molecular allergens). In particular, it is worth mentioning his papers on

• the relevance of defining allergy phenotypes for an appropriate use of anti-allergic drugs and immunotherapy (*Allergy*. 1997;52:693-694; *Ann Allergy Asthma*. 2001;87:48-51);

• the modulatory effects of Nerve Growth Factor and Thymosin beta-4 on allergic inflammation and tissue remodelling (*Int Archs Allergy Immunol*. 2003;131:80-84; *Mol Vis*. 2006;12:1594-600);

• the predictive value of allergy and pulmonary function tests for the diagnosis of asthma in athletes (*Allergy*. 2007;62:1166-1170);

• the ARIA Guidelines for athletes (*Allergy*. 2006;61:681-692) and the methodology for their development (*Allergy*. 2008;63:38-46).

Having an allergist at the guide of EMA is of tremendous importance for calling attention of regulatory bodies on allergic and immunologic diseases, as well as for dealing with the problems related to the increasing use of biologicals and the changing regulations on allergen immunotherapy.

## References

[B1] Noon L Prophylactic inoculation against hayfever Lancet 1911 5 1572

[B2] Commemorating 100 years of allergen immunotherapy J Allergy Clin Immunol 2011 5 3 101 10.1016/j.jaci.2010.11.032 21211638

[B3] 100 years allergen specific immunotherapy Allergy 2011 5 709 809 10.1111/j.1398-9995.2011.02595.x 21518373

[B4] Kaul S May S Luttkopf D Vieths S Regulatory environment for allergen-specific immunotherapy Allergy 2011 5 753 764 10.1111/j.1398-9995.2011.02552.x 21288251

[B5] Shekelle PG Woolf SH Eccles M Grimshaw J Clinical guidelines: developing guidelines BMJ 1999 5 593 596 10.1136/bmj.318.7183.593 10037645 PMC1115034

[B6] Bousquet J Lockey R Malling HJ Allergen immunotherapy: therapeutic vaccines for allergic diseases. A WHO Position Paper J Allergy Clin Immunol 1998 5 558 562 10.1016/S0091-6749(98)70271-4 9802362

[B7] Canonica GW Baena-Cagnani CE Bousquet J Recommendations for standardization of clinical trials with allergen specific immunotherapy for respiratory allergy. A statement of the World Allergy Organization (WAO) Task Force Allergy 2007 5 317 324 10.1111/j.1398-9995.2006.01312.x 17298350

[B8] Canonica GW Bousquet J Casale T Sub-lingual immunotherapy. WAO Position Paper Allergy 2009 5 suppl 91 51 59 10.1111/j.1398-9995.2009.02309.x 20041860

[B9] Malling HJ Weeke B EAACI Immunotherapy position paper Allergy 1993 5 suppl 14 9 458342741

[B10] Cox LS Nelson H Lockey R Allergen immunotherapy. A Practice Parameter. Third update J Allergy Clin Immunol 2011 5 suppl S1 S55 21122901 10.1016/j.jaci.2010.09.034

[B11] Calderon MA Gerth van Wijk R Eichler I Perspectives on allergen-specific immunotherapy in childhood: an EAACI position statement Pediatr Allergy Immunol 2012 5 300 306 10.1111/j.1399-3038.2012.01313.x 22594930

[B12] Bousquet PJ Brozek J Bachert C The CONSORT statement checklist in allergen specific immunotherapy. A GA2LEN Paper Allergy 2009 5 1737 1745 10.1111/j.1398-9995.2009.02232.x 19860788

[B13] Bousquet PJ Calderon MA Demoly P The CONSORT statement applied to allergen-specific immunotherapy with inhalant allergens J Allergy Clin Immunol 2011 5 30 38 10.1016/j.jaci.2010.08.024 21112079

[B14] Frankland AW Augustin R Prophylaxis of summer hayfever and asthma: controlled trial comparing crude grass pollen extracts with isolated main protein component Lancet 1954 5 1055 13164324 10.1016/s0140-6736(54)91620-7

[B15] Kaul S Englert L May S Vieths S Regulatory aspects of specific immunotherapy in Europe Curr Opin Allergy Clin Immunol 2010 5 594 602 10.1097/ACI.0b013e32833fd5d2 20864886

[B16] Lorenz AR Luttkopf D Seitz R Vieths S The regulatory system in Europe with special emphasis on allergen products Int Arch Allergy Immunol 2008 5 263 275 10.1159/000146074 18648190

[B17] Bonini S Tomino C Manual of Clinical Trials in Allergy and Clinical Immunology 2009 Tipografia Cardoni, Rome

[B18] Tomino C New perspectives and new challenges in clinical trial regulation in Italy Ann Ist Sup Sanita 2011 5 19 21 10.4415/ANN_11_01_05 21430333

[B19] Kroon AM The EAMG position on the regulation of existing products for treatment with special reference to named-patient products Arch Paul Ehlrich Inst Bundesamt Sera Impfstoffe Frankf A M 2003 5 7 14 15119017

[B20] Godicke V Hundt F Registration trials for specific immunotherapy in Europe: advanced guidance from the new European Medical Agency guideline Allergy 2010 5 1499 1505 10.1111/j.1398-9995.2010.02436.x 20608914

[B21] Cox L Esch RE Corbett M Hankin C Nelson M Plunkett G Allergen immunotherapy practice in the United States: guidelines, measures, and outcomes Ann Allergy Asthma Immunol 2011 5 289 300 10.1016/j.anai.2011.06.018 21962088

[B22] Cox L Jacobsen L Comparison of allergen immunotherapy practice patterns in the United States and Europe Ann Allergy Asthma Immunol 2009 5 451 460 10.1016/S1081-1206(10)60259-1 20084837

[B23] Bonini S Rasi G Who benefits from immunotherapy? Allergy 1997 5 693 694 10.1111/j.1398-9995.1997.tb01224.x 9265982

[B24] Bonini S Rasi G Torre A D'Amato M Matricardi P The heterogeneity of allergic phenotypes: genetic and environmental interactions Ann Allergy Asthma Immunol 2001 5 suppl 3 48 51 11770684 10.1016/s1081-1206(10)62341-1

[B25] Eichler I Soriano S Close collaboration between academia, industry and drug regulators is required in development of allergen products for specific immunotherapy in children Allergy 2011 5 999 1004 10.1111/j.1398-9995.2011.02582.x 21426358

